# Novel exomphalos genetic mouse model: The importance of accurate phenotypic classification

**DOI:** 10.1016/j.jpedsurg.2013.04.010

**Published:** 2013-10

**Authors:** Helen Carnaghan, Tom Roberts, Dawn Savery, Francesca C. Norris, Conor J. McCann, Andrew J. Copp, Peter J. Scambler, Mark F. Lythgoe, Nicholas D. Greene, Paolo DeCoppi, Alan J. Burns, Agustino Pierro, Simon Eaton

**Affiliations:** a UCL Institute of Child Health, London, UK; b Centre for Advanced Biomedical Imaging, University College London, London, UK; c Centre for Mathematics and Physics in the Life Sciences and Experimental Biology, University College London, London, UK; d Division of Paediatric Surgery, The Hospital for Sick Children, Toronto, Ontario, Canada

**Keywords:** Exomphalos, Gastroschisis, Abdominal wall defect, Rodent model, *Scrib*, *In amnio* micro-MRI, Interstitial cells of Cajal

## Abstract

**Background:**

Rodent models of abdominal wall defects (AWD) may provide insight into the pathophysiology of these conditions including gut dysfunction in gastroschisis, or pulmonary hypoplasia in exomphalos. Previously, a Scribble mutant mouse model (*circletail*) was reported to exhibit gastroschisis. We further characterise this AWD in Scribble knockout mice.

**Method:**

Homozygous *Scrib* knockout mice were obtained from heterozygote matings. Fetuses were collected at E17.5–18.5 with intact amniotic membranes. Three mutants and two control fetuses were imaged by *in amnio* micro-MRI. Remaining fetuses were dissected, photographed and gut length/weight measured. Ileal specimens were stained for interstitial cells of Cajal (ICC), imaged using confocal microscopy and ICC quantified.

**Results:**

127 fetuses were collected, 15 (12%) exhibited AWD. Microdissection revealed 3 mutants had characteristic exomphalos phenotype with membrane-covered gut/liver herniation into the umbilical cord. A further 12 exhibited extensive AWD, with eviscerated abdominal organs and thin covering membrane (intact or ruptured). Micro-MRI confirmed these phenotypes. Gut was shorter and heavier in AWD group compared to controls but morphology/number of ICC was not different.

**Discussion:**

The Scribble knockout fetus exhibits exomphalos (intact and ruptured), in contrast to the original published phenotype of gastroschisis. Detailed dissection of fetuses is essential ensuring accurate phenotyping and result reporting.

Abdominal wall defects include exomphalos and gastroschisis both of which result in differing pathophysiological consequences such as gut dysfunction in gastroschisis and pulmonary hypoplasia in exomphalos. The cause of these sequelae is not fully understood and of great research interest. Genetic rodent models of abdominal wall defects could provide a useful tool in further understanding these conditions. However, phenotyping can be difficult and requires detailed microdissection to ensure accurate result interpretation and reporting.

## 1 Background

Ventral abdominal wall defects (AWD) are relatively common and include exomphalos (2.5/10,000 live births) [1] and gastroschisis (4.4/10,000 live births) [2]. Both have a significant clinical impact and distinct phenotypic appearances. Exomphalos is characterised by a ventral wall defect disrupting the umbilical ring resulting in herniation of abdominal viscera including gut and liver into the base of the umbilical cord, contained within a membranous sac. The condition is frequently associated with other abnormalities including pulmonary hypoplasia, structural defects of the heart, diaphragm and limbs, and metabolic and chromosomal disorders. However, gut dysfunction and structural gut defects are rare in exomphalos [3]. On the other hand, gastroschisis is a paraumbilical ventral wall defect, usually (95%) lying to the right of the umbilicus [4], through which bowel and very rarely other organs herniate. Characteristically the peritoneal sac is deficient, placing the bowel in direct contact with the irritant amniotic fluid. Gastroschisis is rarely associated with extra-intestinal abnormalities and the most significant cause of morbidity is that of gut dysfunction, which may require prolonged parenteral feeding and carries the risk of central line infections, sepsis and liver dysfunction [5].

The cause, development and pathophysiology of such conditions still remain controversial and are subject to great research interest. For example, it is not clear why gut dysfunction is found mainly in gastroschisis [6–9] while pulmonary hypoplasia is a characteristic feature of exomphalos [10,11]. Investigating abdominal wall defects (AWD) in robust animal models is essential to further our pathophysiological knowledge and develop improved treatment options. Genetic rodent models are advantageous owing to the short gestational period, similar sequence of abdominal wall closure with resolution of physiological hernia by day 16.5 gestational age [12], large litter sizes and early development of the defect without the need for surgical creation. However, characterization of AWD in rodents is not always accurate and currently there is only one genetic animal model of isolated gastroschisis (mice lacking aortic carboxypeptidase-like protein [ACLP]) reported in the literature [9]. Of note, the ventral wall defect present in the *Alx4^-/-^* mutant mouse was originally reported in the literature as gastroschisis [13] and then subsequently as exomphalos [14].

Previously, the *circletail* mouse mutant, which carries a single base insertion in the *Scrib* (*scribbled homolog*) gene resulting in a frame shift and premature termination of the Scribble protein, was reported to exhibit gastroschisis in association with craniorachischisis [15]. Subsequently, the Scribble mutant model has been further developed by production of a floxed allele, enabling conditional gene targeting and analysis of *Scrib* gene function in various biological systems [16,17]. Our aim was to fully characterize the AWD that results from Scribble loss of function, using the floxed allele to generate *Scrib* null fetuses.

## 2 Materials and methods

All experimental protocols were granted Home Office approval under the UK Animals (Scientific Procedures) Act 1986.

### 2.1 Animals

The floxed *Scrib* (*Scrib^fl^*) allele was a kind gift from Dr Patrick Humbert. Matings between *Scrib^fl/fl^* homozygotes and mice expressing the ubiquitously expressed βactin-Cre generated a null allele (*Scrib^-^*). Timed matings between heterozygotes (*Scrib^fl/-^*) generated null fetuses (*Scrib^-/-^*). Pregnant mothers were euthanized by cervical dislocation at two gestational stages: (1) day 17.5 (E17.5) when amniotic fluid volume is greater to aid dissection of the fetus from the amniotic cavity and abdominal wall phenotyping; (2) day 18.5 (E18.5) just before full term for gut length, weight and cellular phenotyping. The fetuses were collected by maternal hysterectomy under a dissecting microscope. The uterine muscle was carefully removed leaving the amniotic membranes intact and the intra-amniotic fetus was photographed under a stereo microscope. The fetuses were then carefully dissected from the amniotic sac, euthanized by cervical dislocation and photographed under a stereo microscope and placed on ice. In randomly selected E18.5 normal and mutant fetuses, the intestine was removed from the gastroesophageal junction to the ileocaecal valve. Gut length (cm) and weight (mg) were measured and weight per unit length was calculated. Data are mean ± standard error of the mean and were compared using t-tests. Randomly selected fetuses were genotyped by polymerase chain reaction (PCR) using DNA from tail biopsies and primers as previously described [17].

### 2.2 In amnio micro-MRI

Phenotypically normal and mutant fetuses were imaged within intact amniotic membranes. Fetuses were imaged at E17.5 when the physiological hernia has resolved but the amniotic fluid volume is still relatively large to aid visualisation of fine intra-amniotic structures. Intra-amniotic fetuses were fixed in 4% paraformaldehyde (PFA) and then embedded in agarose. T2-weighted, high-resolution (256^3^ pixels with a 25.6^3^ mm field of view; resolution of 100 μm/pixel) micro-MR images were acquired using a 3D fast spin echo sequence. Images were reconstructed using ImageJ (NIH, USA) and visualised using Amira 5.4 (Visage Imaging, Inc., Berlin, Germany).

### 2.3 Interstitial cells of Cajal immunohistochemistry and microscopy

Within the ACLP knockout mouse gastroschisis model a reduction in number of bowel wall interstitial cells of Cajal (ICC) has been found within the eviscerated small intestine compared to non-eviscerated small intestine and normal phenotype controls [9]. As such, as an additional way to determine whether the *Scrib^-/-^* ventral wall phenotype is that of gastroschisis or exomphalos, eviscerated ileal gut specimens were evaluated for ICC numbers and compared with controls.

Gut specimens were collected from normal controls and fetuses with AWD (large ventral wall defect phenotype) near full term at E18.5. Whole mount ileal specimens were prepared by longitudinally opening the ileum and stripping the mucosa (reducing tissue autofluorescence and mast cell numbers) from the muscularis leaving the circular and longitudinal muscles intact. Specimens were fixed in 4% PFA. Interstitial cells of Cajal (ICC) were labelled with c-kit (R&D Systems® mouse SCF R/c-kit affinity purified polycolonal antibody raised in goat), the most commonly used ICC marker, and secondarily stained with donkey anti-goat IgG (Molecular Probes®, Alexa Fluor® 488). Nuclei were labelled with DAPI (4′,6-Diamidino-2-phenylindole dihydrochloride, Molecular Probes®). Specimens were mounted flat and oriented with circular smooth muscle uppermost on microscopy slides. Ten high powered fields (40 × objective) were imaged per specimen using confocal microscopy (Zeiss LSM 710) creating full thickness, z stack image sets. ImageJ cell counter software was used to manually count all ICC present in each z-stack. Mast cells also stain positive for c-kit but are morphologically distinct from ICC and were therefore identified and excluded during cell counting. Specimens were allocated a randomly selected code and ICC counted on coded specimens so that the researcher counting cells was blinded to the macroscopic phenotype.

## 3 Results

### 3.1 Characterisation of Scribble null mice

A total of 127 fetuses (E18.5 n = 78, E17.5 n = 49) were collected: 112 were phenotypically normal and 15 (12%) exhibited an AWD of which 5 occurred in isolation and 10 with coexisting craniorachischisis. None of the fetuses exhibited an isolated neural tube defect. Within this study group 58 fetuses were genotyped: 12 (21%) were ‘wild type’ (*Scrib^fl/fl^*; all phenotypically normal), 20 (34%) were heterozygotes (*Scrib^fl/-^*; 19 normal and 1 with coexisting large AWD and craniorachischisis) and 26 (45%) were homozygotes (*Scrib^-/-^*; 20 normal, 4 with isolated AWD [3 with large AWD and 1 with small AWD] and 2 with coexisting large AWD and craniorachischisis). Hence, the penetrance of AWD in homozygous *Scrib^-/-^* mice is 23%.

### 3.2 Abdominal wall defect morphology

**Fig. 1:**
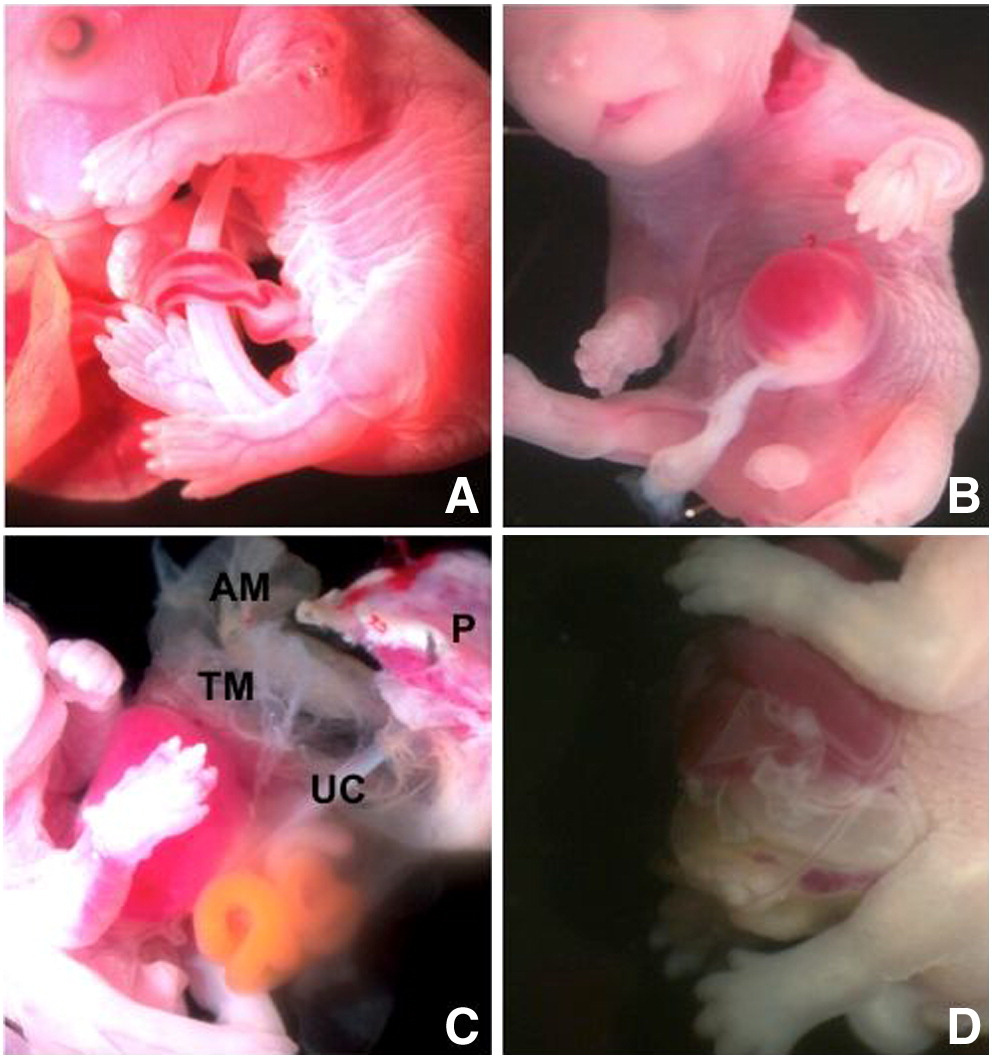
*Scrib* mutant mouse abdominal wall phenotypes at E17.5, magnification × 8. A. Normal phenotype with intact abdominal wall and normal umbilical cord insertion. B. Exomphalos with membrane covered liver and gut herniation into the base of the umbilical cord. C. Extensive ventral abdominal wall defect with evisceration of liver, gut and spleen. A ruptured thin membrane (TM) is present associated with the abdominal viscera and exhibits vascular attachments to the amniotic membrane (AM). The placenta (P) and umbilical cord (UC) have been left intact. D. Large ventral wall defect with intact thin membrane covering herniated abdominal viscera (superior pole of the membrane was iatrogenically ruptured during dissection).

Dissection under a stereo microscope revealed normal fetuses and two types of ventral abdominal wall pathology. Fetuses classified as having normal phenotype exhibited an intact ventral wall with correct umbilical cord insertion (Fig. 1A). The first type of abdominal wall defect was characteristic of exomphalos (total n = 3; E18.5 n = 1, E17.5 n = 2): the ventral abdominal wall defect disrupted the umbilical ring, and there was herniation of bowel and liver, extending into the base of the umbilical cord and contained within a membranous sac (Fig. 1B). The second pathological defect comprised a complete failure of ventral abdominal wall closure (total n = 12, E18.5 n = 8, E17.5 n = 4) resulting in exteriorisation of abdominal viscera including bowel, stomach, spleen and liver. In these fetuses, there was also evidence of a thin, ruptured membrane covering the viscera with vascular connections to the amniotic membrane (Fig. 1C). Because of the fine nature of the membrane, this rupture may have either occurred antenatally or iatrogenically during dissection of the fetus from the amniotic membrane. Following dissection of one such mutant at E17.5, a fine intact membrane covering the herniated abdominal viscera, and in continuity with the umbilical cord, was evident (Fig. 1D). Also of note, the umbilical cord and placenta were normal. Dissection of the fetus from the amniotic cavity was easier at E17.5 owing to the larger volume of amniotic fluid enabling identification and preservation of the fine membrane covering the herniated viscera. The abdominal wall phenotypes were the same at both E17.5 and E18.5 developmental stages. Finally, the co-existent neural tube defect was that of craniorachischisis (as previously described [15]) in which there was gross failure of neural tube closure resulting in almost the entire brain and spinal cord being exposed. No anomalies of the limbs or digits were present.

### 3.3 In amnio micro-MRI

**Fig. 2:**
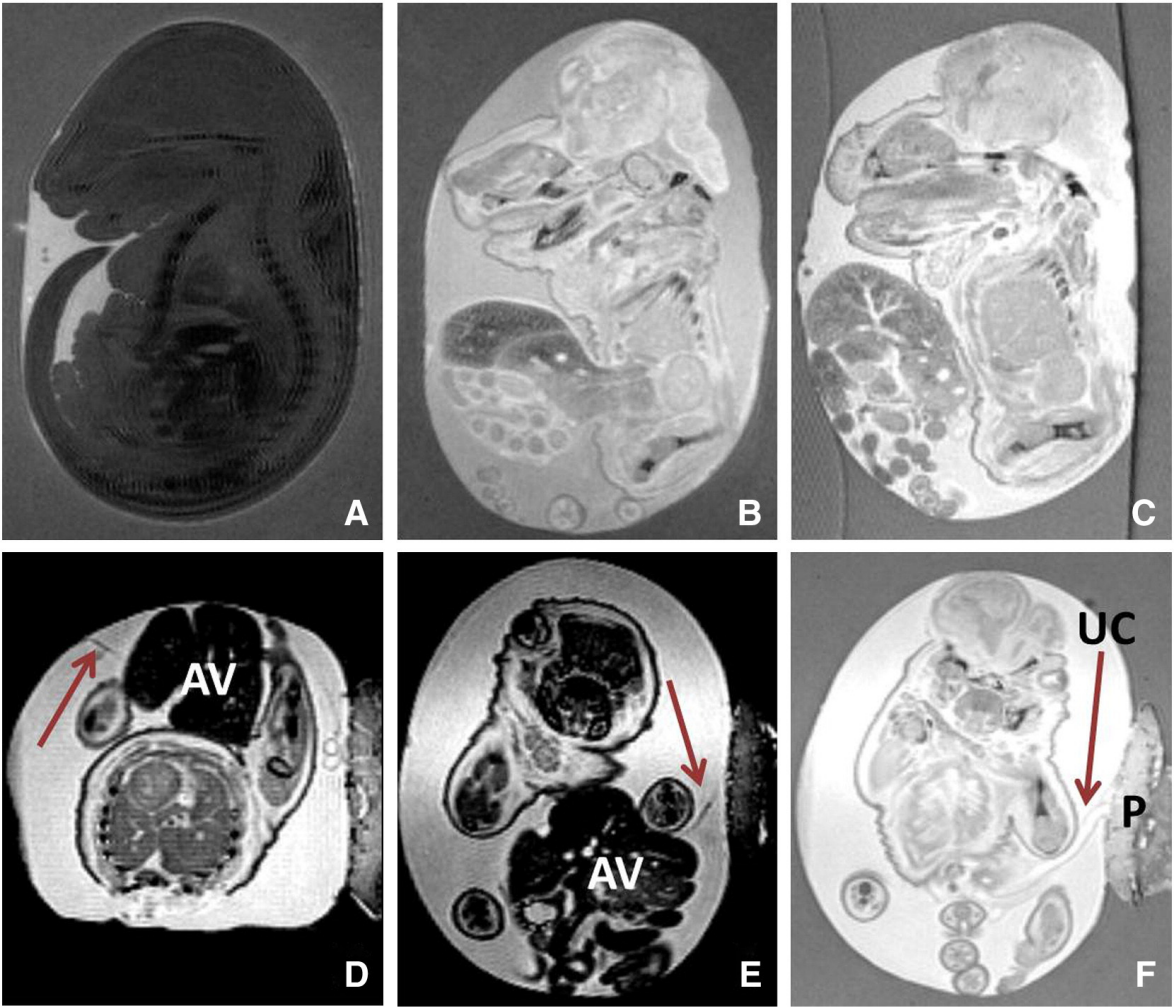
*In amnio* micro-MRI of E17.5 *scrib* mutant mice. A. Sagittal image of the normal phenotype. B. Sagittal image of exomphalos showing abdominal viscera herniated within an intact membrane. Craniorachischisis is evident with extensive failure of neural tube closure from cranium to sacrum. C. Sagittal image of the large ventral abdominal wall defect showing eviscerated abdominal contents with no discernable membrane covering. Craniorachischisis is again visualised. D. Contrast-enhanced axial image of the large ventral abdominal wall defect with evidence of a membranous structure (arrow) associated with the externalised abdominal viscera (AV). E. Coronal image of the large ventral abdominal wall defect also revealing a membranous structure (arrow) in association with the externalised abdominal viscera (AV). F. Coronal image (increased image contrast) of the large ventral abdominal wall defect showing the presence of a normal umbilical cord (UC) and placenta (P).

MRI of two phenotypically normal mice showed a complete abdominal wall and normal insertion of the umbilical cord (Fig. 2A). Imaging of one characteristic exomphalos phenotype (described above) showed disruption of the umbilical ring and herniation of bowel and liver within a membrane (Fig. 2B). MR images of two mutants with complete failure of ventral wall closure showed the externalised abdominal viscera to be free floating within the amniotic fluid (Fig. 2C). However, post-processing contrast enhancement of these images revealed a fine membranous structure associated with the abdominal viscera and extending from the abdominal wall to the amniotic membrane (Fig. 2D–E). This confirmed the presence of a membrane (intact or ruptured) covering the abdominal viscera in this phenotype. Also of note is the presence of a normal placenta and normal umbilical cord (Fig. 2F). In addition, the coexistent craniorachischisis was clearly visualised and failure of neural tube closure evident from the cranium to sacrum (Figs. 2B–C). Finally, identification of fine membraneous structures was aided by the bright contrast provided by the amniotic fluid and relatively large volume of amniotic fluid surrounding the fetus.

### 3.4 Gross intestinal morphology

The bowel of fetuses with the large ventral wall defect was observed to be shorter and more tortuous than normal phenotypes, with no evidence of peel. There was a significant and marked difference in intestinal length between normal phenotype mice (7.21 ± 0.12 cm; n = 15) and large AWD (5.06 ± 0.16 cm; n = 8; *p* < 0.0001). However, there was a small difference in weight per unit gut length which just reached statistical significance (normal phenotype: 10.3 ± 0.9 mg/cm versus large AWD: 11.4 ± 0.57 mg/cm; *p* = 0.0453).

### 3.5 Interstitial cells of Cajal

**Fig. 3:**
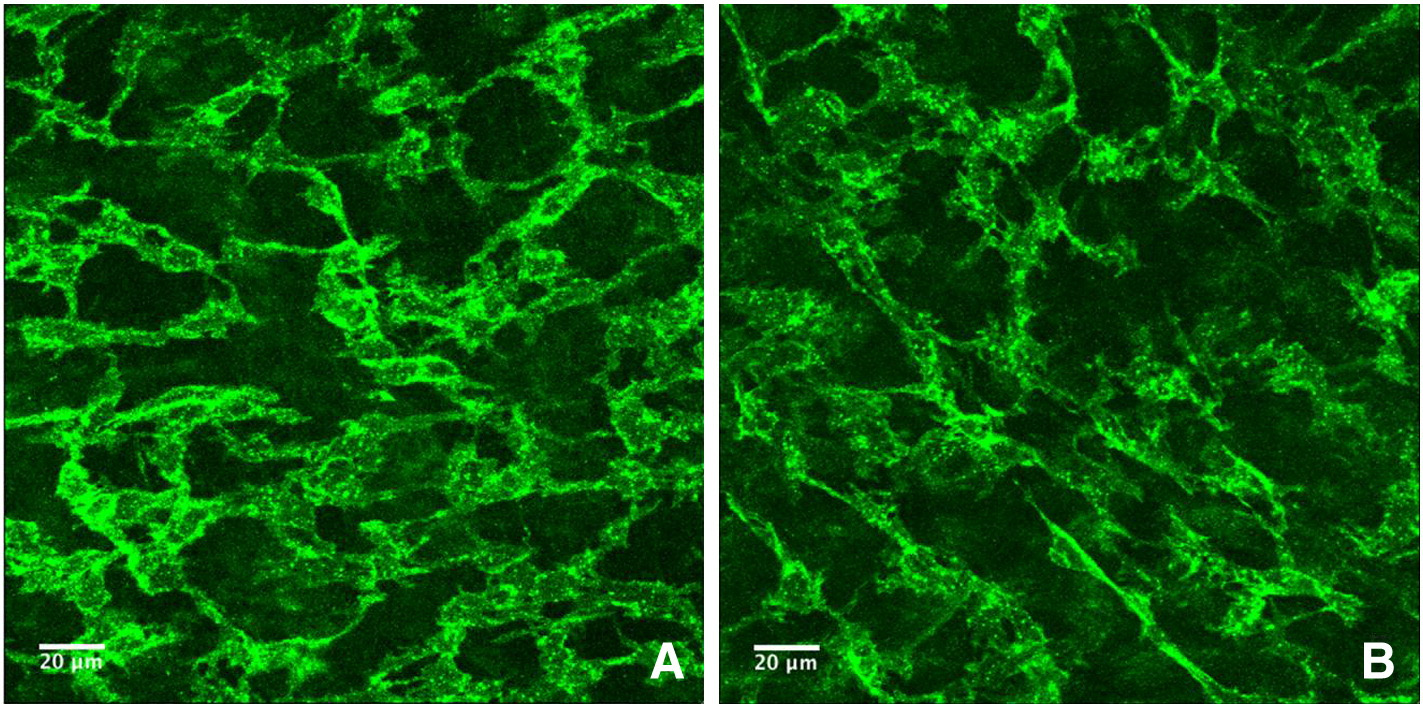
Whole mount E18.5 ileal specimens stained for interstitial cells of Cajal with c-kit, maximum intensity project of z-stacks acquired by confocal microscopy, objective 40 × (45 nm^2^). A. Specimen from normal phenotype mouse, mean number of ICC per high powered field was 129, range 105–146. B. Specimen from large abdominal wall defect phenotype, mean number of ICC per high powered field was 193, range 119–232.

ICC at the level of the myenteric plexus (ICC-MY) in both the AWD (n = 3) and control groups (n = 6) were well developed exhibiting numerous branching cytoplasmic processes that interconnect with their counterparts creating a mature plexus. ICC at the level of the deep muscular plexus (ICC-DMP) were not observed in either group, as expected for this gestational stage. Within the control group the mean number of ICC per high powered field (45 nm^2^) was 129, range 105–146 (Fig. 3A). In comparison, the mean number of ICC per high powered field for the mutant group was 193, range 119–232 (Fig. 3B). Although the number of ICC within the mutant group appears higher than the control group the difference was not statistically significant.

## 4 Discussion

Contrary to the original description of the *circletail* (*Scrib^crc^*) mutant mouse as a model of gastroschisis [15], null fetuses derived from the floxed *Scrib* allele exhibit an exomphalos phenotype of varying severity with intact and ruptured membranes. The least severe form is a characteristic exomphalos phenotype with herniated bowel and liver covered by a membranous sac. The most severe form is a more ambiguous large ventral body wall defect with a thin membrane covering the exteriorised abdominal viscera including bowel, stomach, liver and spleen, which may rupture intra-amniotically. This is similar to the situation with the *Alx4* mutant mouse, which was originally described to exhibit gastroschisis [13] but was later reported to exhibit exomphalos [14]. This highlights the difficulties of accurate phenotyping of AWD in rodent models.

Delineation of the large exomphalos phenotype was possible through careful dissection and preservation of the thin covering membrane and other structures including the umbilical cord and placenta under the dissecting stereo microscope. High-resolution *in amnio* MRI enabled visualisation and confirmation of this thin covering membrane which provides evidence that the observed defect is in fact a large exomphalos rather than an atypical, large gastroschisis. Also, the presence of herniated liver in both *Scrib* and *Alx4* mutant fetuses provides further confirmation that this mutant is exomphalos rather than gastroschisis, which is very rarely associated with externalised liver. In addition, *Scrib* mutant fetuses were found to have a normal placenta and umbilical cord providing evidence against this being a body stalk defect. In humans, body stalk defects comprise a very rare, large AWD with evisceration of abdominal and/or thoracic organs, an absent or extremely short umbilical cord and associations with head, face and limb anomalies [18,19].

Correct phenotyping is essential given that exomphalos and gastroschisis differ greatly in terms of their associated pathologies and morbidities. For example, the most significant morbidity in gastroschisis is gut dysfunction [5], the cause of which is poorly understood but is hypothesised to result from reduced numbers and poorly developed ICC [7–9], which are important in the initiation and propagation of peristalsis, in eviscerated gut. In turn, ICC disruption is thought to be secondary to the gut being exposed and in direct contact with the irritant amniotic fluid causing gut inflammation, which in humans creates thickened, foreshortened, matted bowel with a peel covering. As such, investigating gastroschisis-associated gut dysfunction within a mutant rodent model requires accurate AWD phenotyping. Otherwise, experimental data could be collected from a mislabelled mutant model of exomphalos with membrane covered gut herniation resulting in inaccurate reporting of gastroschisis research data. However, the *Scrib* mutant mouse would be a useful model to investigate the pathophysiology of pulmonary hypoplasia associated with exomphalos, which is a significant cause of morbidity within this patient group. A strong association has been shown between pulmonary hypoplasia and major/giant exomphalos defects [20,21]. As such, the *Scrib* mutant mouse is advantageous for the investigation of this condition given this model exhibits both small and giant defect sizes enabling direct comparison of lung development between extremes of exomphalos phenotypes as well as normal controls. Characterisation of both macroscopic and microscopic lung development would be possible. In addition, in-utero therapy to improve lung development could potentially be investigated.

The large exomphalos defect we observed in Scribble null fetuses was associated with a shorter gut length compared to normal controls. Although the large exomphalos defect mice displayed a slightly greater weight per unit length of gut, this difference was not marked and only just achieved statistical significance. Further assessment of these phenomena could be undertaken through analysis of hematoxylin and eosin stained cross-section gut to determine muscle wall thickness and villus height. The differences seen may suggest a global disruption of bowel development as part of the AWD formation. On the other hand, it could represent membrane rupture placing the bowel in direct contact with the amniotic fluid resulting in the beginnings of gut inflammation as described above for gastroschisis. Even if the latter theory is true, the timing of membrane rupture is unknown and may differ between fetuses. Unless one was able to serially assess the intactness of this membrane prenatally *in vivo*, this model could not be used to reliably mimic and investigate the impact of amniotic fluid exposure on structural or functional gut development as seen in gastroschisis. In addition, the preliminary data presented here show that ICC are normally developed compared to controls which contradicts the gastroschisis ICC research discussed above providing evidence that the membrane may be intact in these sampled mutants. We used an anti-c-kit antibody to detect ICC in whole mount specimens. Although c-kit also labels mast cells, the majority of these are located within the mucosa, which was removed during preparation of our whole mount specimens. In addition, mast cells have a distinctly different architecture compared to ICC and are easily identified on whole mount sections during manual cell counting. An alternative approach would have been to use other antibodies for ICC, such as Ano1 [22].

It appears that the terms exomphalos and gastroschisis are being used interchangeably within the literature to describe genetic rodent AWD models. Characterisation of such defects can be challenging but identification of umbilical cord insertion, location of the ventral wall defect, effect on the umbilical ring and presence or absence of a covering membrane through careful microdissection is the key to accurate phenotyping. In addition, phenotyping can be greatly aided with the use of novel imaging methodologies such as *in amnio* micro-MRI.

## References

[1] Weber T.R., Au-Fliegner M., Downard C.D. (2002). Abdominal wall defects. Curr Opin Pediatr.

[2] Kilby M.D. (2006). The incidence of gastroschisis: Is increasing in the UK, particularly among babies of young mothers. BMJ.

[3] Vachharajani A.J., Rao R., Keswani S. (2009). Outcomes of exomphalos: an institutional experience. Pediatr Surg Int.

[4] Sadler T.W. (2010). The embryologic origin of ventral body wall defects. Semin Pediatr Surg.

[5] Bradnock T.J., Marven S., Owen A. (2011). Gastroschisis: one year outcomes from national cohort study. BMJ.

[6] Morrison J.J., Klein N., Chitty L.S. (1998). Intra-amniotic inflammation in human gastroschisis: possible aetiology of postnatal bowel dysfunction. Br J Obstet Gynaecol.

[7] Midrio P., Vannucchi M.G., Pieri L. (2008). Delayed development of interstitial cells of Cajal in the ileum of a human case of gastroschisis. J Cell Mol Med.

[8] Midrio P., Faussone-Pellegrini M.S., Vannucchi M.G. (2004). Gastroschisis in the rat model is associated with a delayed maturation of intestinal pacemaker cells and smooth muscle cells. J Pediatr Surg.

[9] Danzer E., Layne M.D., Auber F. (2010). Gastroschisis in mice lacking aortic carboxypeptidase-like protein is associated with a defect in neuromuscular development of the eviscerated intestine. Pediatr Res.

[10] Danzer E., Hedrick H.L., Rintoul N.E. (2010). Assessment of early pulmonary function abnormalities in giant omphalocele survivors. J Pediatr Surg.

[11] Kamata S., Usui N., Sawai T. (2008). Prenatal detection of pulmonary hypoplasia in giant omphalocele. Pediatr Surg Int.

[12] Brewer S., Williams T. (2004). Finally, a sense of closure? Animal models of human ventral body wall defects. Bioessays.

[13] Qu S., Niswender K.D., Ji Q. (1997). Polydactyly and ectopic ZPA formation in Alx-4 mutant mice. Development.

[14] Matsumaru D., Haraguchi R., Miyagawa S. (2011). Genetic analysis of hedgehog signalling in ventral body wall development and the onset of omphalocele formation. PLoS One.

[15] Murdoch J.N., Henderson D.J., Doudney K. (2003). Disruption of *scribble* (Scrb1) causes severe neural tube defects in the circletail mouse. Hum Mol Genet.

[16] Hartleben B., Widmeier E., Wanner N. (2012). Role of the polarity protein *scribble* for podocyte differentiation and maintenance. PLoS One.

[17] Pearson H.B., Perez-Mancera P.A., Dow L.E. (2011). SCRIB expression is deregulated in human prostate cancer, and its deficiency in mice promotes prostate neoplasia. J Clin Invest.

[18] Bugge M. (2012). Body stalk anomaly in Denmark during 20 years (1970-1989). Am J Med Genet.

[19] Paul C., Zosmer N., Jurkovic D. (2001). A case of body stalk anomaly at 10 weeks of gestation. Ultrasound Obstet Gynecol.

[20] Argyle J.C. (1989). Pulmonary hypoplasia in infants with giant abdominal wall defects. Pediatr Pathol.

[21] Charlesworth P., Ervine E., McCullagh M. (2009). Exomphalos major: the Northern Ireland experience. Pediatr Surg Int.

[22] Gomez-Pinilla P.J., Gibbons S.J., Bardsley M.R. (2009). Ano1 is a selective marker of interstitial cells of Cajal in the human and mouse gastrointestinal tract. Am J Physiol Gastrointest Liver Physiol.

